# Immunohistochemistry for Skin Cancers: A Comprehensive Approach to the Diagnosis of Squamous Cell Carcinoma

**DOI:** 10.3390/cancers17101629

**Published:** 2025-05-12

**Authors:** Vlad-Mihai Voiculescu, Radu-Marian Marinescu, Sorin Dutulescu, Florica Stăniceanu

**Affiliations:** 1 Department of Dermatology, “Carol Davila” University of Medicine and Pharmacy, 050474 Bucharest, Romania; vlad.voiculescu@umfcd.ro (V.-M.V.); sorin.dutulescu@drd.umfcd.ro (S.D.); floriastaniceanu@yahoo.fr (F.S.); 2 Elias Emergency University Hospital, 011461 Bucharest, Romania; 3 Pathology Department, “Matei Bals” National Institute for Infectious Diseases, 021105 Bucharest, Romania

**Keywords:** squamous cell carcinoma, immunohistochemistry, skin cancer

## Abstract

Background: Squamous cell carcinoma (SCC) is a heterogeneous group of epithelial malignancies with varied morphologies and clinical behaviors. While histopathology is the diagnostic gold standard, it can be limited in distinguishing SCC from morphologic mimics. Immunohistochemistry (IHC) has therefore become a critical adjunct, enhancing diagnostic accuracy and providing prognostic insights. Objective: This narrative review aims to evaluate the diagnostic, differential, and prognostic roles of commonly used IHC markers in SCC, with particular emphasis on their utility in distinguishing SCC from histologic mimickers across different anatomical sites. Methods: One hundred and five peer-reviewed articles were analyzed for their relevance to the immunohistochemical characterization of SCC. Markers were categorized based on their diagnostic function, role in differential diagnosis, and prognostic value. Results: Key markers such as p40, p63, CK5/6, and DSG3 consistently demonstrated high sensitivity and specificity for SCC, reinforcing their value in confirming squamous differentiation. Conversely, exclusion markers like Ber-EP4, CK7, TTF-1, S100, and SOX10 were essential in ruling out basal cell carcinoma, adenocarcinoma, and melanoma. Additionally, markers such as Ki-67, p16, and CD44 offered prognostic information regarding tumor aggressiveness, HPV status, and therapy response. These findings confirm the critical role of IHC not only in diagnosing SCC but also in resolving complex differential diagnoses. Conclusions: IHC markers serve as indispensable tools in the diagnostic workup of SCC, particularly in distinguishing it from other neoplasms with overlapping histologic features. The clear correlation between marker expression and diagnostic categories supports the systematic use of IHC to improve diagnostic precision and inform prognosis. Future integration with molecular diagnostics may further refine personalized treatment approaches in SCC.

## Simple Summary

Squamous cell carcinoma (SCC) encompasses a diverse group of epithelial malignancies with varying morphology, biological behavior, and clinical implications. Although histopathological examination remains the cornerstone of diagnosis, immunohistochemistry (IHC) has become indispensable in confirming squamous differentiation, guiding differential diagnosis, and offering prognostic insight. This systematic review aims to synthesize and organize current evidence on the most frequently used IHC markers in SCC across various anatomical sites, including their diagnostic performance, role in distinguishing mimickers, and utility in staging and risk stratification.

## 1. Introduction

Squamous cell carcinoma (SCC) is a common form of epithelial cancer that shows features of squamous differentiation. It can arise in multiple locations throughout the body, including the skin, mucosal surfaces of the head and neck, esophagus, and cervix, reflecting its broad anatomical distribution. The clinical presentation of SCC varies significantly, with some lesions remaining localized and indolent, particularly on the skin, while others, especially in mucosal sites, exhibit aggressive behavior and a higher propensity for metastasis [1,2,3,4,5,6,7,8].

Historically, SCC diagnosis has been based on histopathological examination using hematoxylin and eosin (H&E) staining. However, this method alone often proves insufficient in specific clinical contexts, such as small biopsies, poorly differentiated tumors, or uncommon histologic variants like basaloid, sarcomatoid, or clear cell SCC. Moreover, conventional morphology lacks the molecular resolution required for accurate tumor stratification or treatment prediction [5,8,9,10,11].

In this context, immunohistochemistry (IHC) has emerged as a key ancillary technique, enabling the identification of lineage-specific proteins, proliferation markers, and stemness indicators in formalin-fixed, paraffin-embedded tissue specimens. Markers such as p40, p63, and CK5/6 have become indispensable in confirming squamous differentiation. Others, including Ki-67, CD44, and p16, provide valuable insights for prognostic stratification and tumor staging. At the same time, exclusion markers like Ber-EP4, CK7, TTF-1, and S100 play an essential role in the differential diagnosis of SCC against entities such as adenocarcinomas, basal cell carcinomas, and melanomas [7,12,13,14,15,16,17,18,19,20].

Despite the widespread use of IHC in SCC diagnostics, there is a lack of structured syntheses that summarize marker performance and specificity across different SCC variants and anatomical contexts. Most publications to date have focused on isolated markers or site-specific subtypes, without offering an integrative diagnostic and prognostic framework.

The aim of this review is to synthesize and systematically organize available evidence regarding the most relevant immunohistochemical markers used in squamous cell carcinoma. We focus on their role in positive diagnosis, differential diagnosis, and prognostic stratification across various SCC subtypes and anatomical sites. Through qualitative synthesis of original studies and case reports, this review intends to provide a practical reference for pathologists and clinicians and to support a more standardized, evidence-based approach in SCC evaluation.

To achieve this, we conducted a structured narrative review based on predefined inclusion and exclusion criteria, focusing on original studies involving immunohistochemical markers applied in SCC diagnosis, differential diagnosis, and staging.

## 2. Materials and Methods

### 2.1. Protocol and Registration

This systematic review was conducted in accordance with the 2020 Preferred Reporting Items for Systematic Reviews and Meta-Analyses (PRISMA) guidelines. The review protocol was not prospectively registered in PROSPERO or any other systematic review registry.

### 2.2. Eligibility Criteria

Inclusion criteria were defined prior to data collection and were as follows:

- Original peer-reviewed articles, clinical case series, or case reports involving immunohistochemical (IHC) analysis in squamous cell carcinoma (SCC);
- Articles published in English between January 2000 and March 2025;
- Studies reporting the diagnostic, differential, or prognostic role of IHC markers in SCC;
- Inclusion of conventional SCC or histologic variants such as basaloid, sarcomatoid, or clear cell subtypes;
- Availability of full text and reporting of marker expression patterns, sensitivity, specificity, or clinical utility.
- Exclusion criteria included the following:
- Articles lacking any immunohistochemical component;
- Narrative reviews, editorials, and expert opinions without original data or methodology;
- Studies focused exclusively on non-squamous malignancies;
- Publications not available in full text or published in languages other than English.

### 2.3. Information Sources and Search Strategy

A systematic literature search was performed across three major databases: PubMed, ScienceDirect, and Google Scholar. The search included articles published up to March 2025. Search terms were structured using Boolean logic and MeSH keywords related to squamous cell carcinoma and immunohistochemistry. Representative search terms included the following:

- “squamous cell carcinoma AND immunohistochemistry”;
- “p40 OR p63 OR CK5/6 AND SCC”;
- “IHC AND squamous cell carcinoma AND prognostic markers”;
- “basaloid SCC AND differential diagnosis”;
- “desmoglein-3 AND SCC”.

Additional articles were retrieved through backward citation tracking of the included studies.

### 2.4. Study Selection

All retrieved records were imported into a shared screening database. Duplicate entries were removed manually. Two independent reviewers (V.M.V. and R.M.M.) screened titles and abstracts for relevance. Full texts of potentially eligible studies were reviewed to determine final inclusion. Disagreements were resolved through discussion with a third reviewer (F.S.).

**Figure 1:**
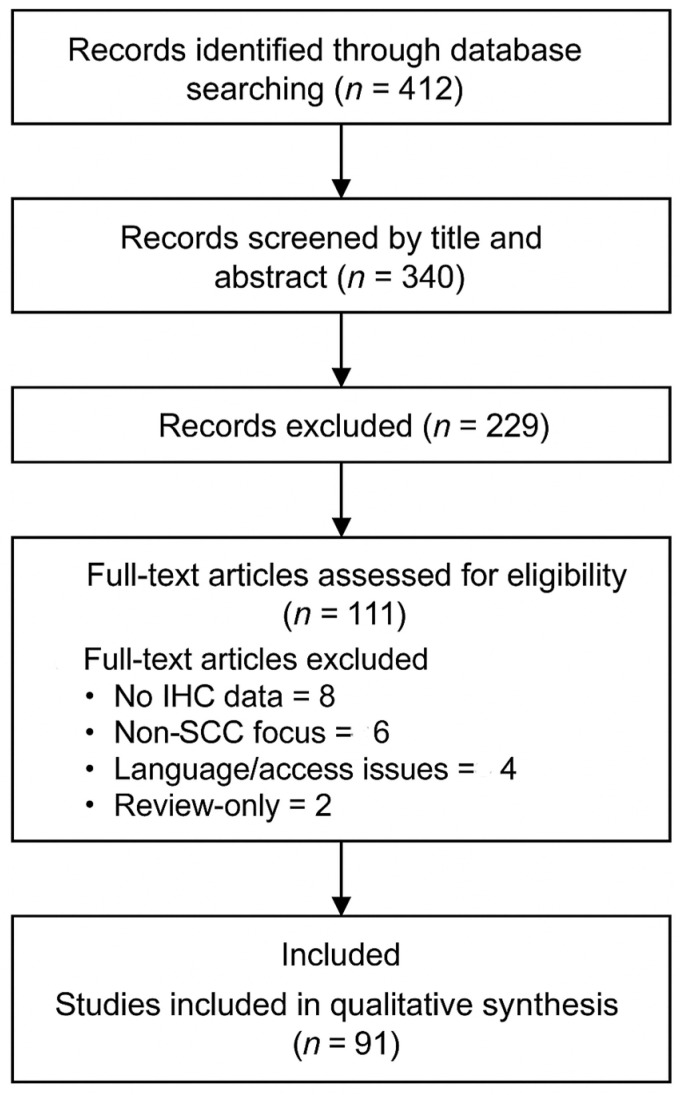
PRISMA 2020 flowchart illustrating the study selection process.

In total, 412 records were identified, of which 72 were duplicates. After title and abstract screening, 229 records were excluded. A total of 111 full-text articles were assessed for eligibility. Of these, 20 were excluded for the following reasons: absence of IHC data (n = 8), focus on non-SCC tumors (n = 6), language or access limitations (n = 4), and lack of original data (n = 2). Finally, 91 studies were included in the qualitative synthesis. The complete selection process is illustrated in the PRISMA 2020 flow diagram (Figure 1).

### 2.5. Data Extraction and Management

A standardized data extraction form was used to capture relevant variables from each included article. The following data were extracted:

- Anatomical site of the tumor;
- SCC subtype (conventional or variant);
- IHC markers assessed;
- Reported sensitivity, specificity, and staining patterns;
- Diagnostic, differential, or prognostic applications.

Data were extracted independently by two reviewers and compiled into comparative tables to facilitate thematic synthesis. No automation tools were used.

### 2.6. Risk of Bias and Quality Assessment

Given the heterogeneity of included studies in terms of design and reporting, no formal risk of bias tool (e.g., QUADAS-2) was applied. Studies were appraised qualitatively based on methodological clarity, cohort size, and completeness of IHC reporting.

### 2.7. Data Synthesis

A qualitative synthesis was performed. Studies were grouped thematically according to the function of the IHC markers: diagnostic confirmation, differential diagnosis from morphologic mimics, and prognostic stratification. Marker-specific sensitivity, specificity, and staining features were summarized narratively and presented in structured summary tables. No meta-analysis or pooled statistical analysis was conducted due to variability in marker targets, tissue sites, and reporting standards.

## 3. Results

### 3.1. Diagnostic Markers Supporting Squamous Differentiation

**Figure 2:**
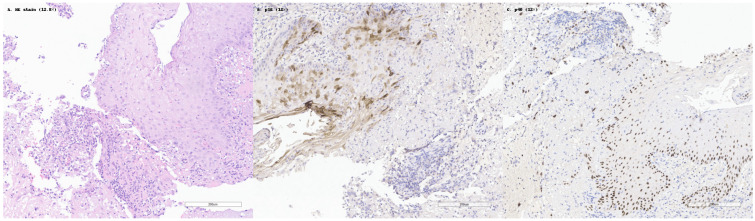
Histopathological and immunohistochemical features of a moderately differentiated squamous cell carcinoma (SCC). (**A**) Hematoxylin and eosin (HE) staining reveals isolated tumor cells and cords of atypical squamous epithelial cells, moderate nuclear pleomorphism, and focal keratinization. (**B**) p16 immunostaining shows complete absence of nuclear and cytoplasmic reactivity in tumor cells, indicating lack of HPV-driven oncogenic involvement. Internal control in some of the intact squamous cell, adjacent to infiltrative component. (**C**) p40 demonstrates strong and diffuse nuclear positivity throughout the dispersed tumor nests, confirming squamous cell lineage and differentiation. Internal control in squamous cells of the basal layers. All panels are shown at original magnifications ranging from 12× to 12.8×, as indicated.

Among the studies analyzed, several immunohistochemical markers emerged as consistently useful in confirming squamous differentiation in SCC. The most frequently reported and validated markers were p40, p63, CK5/6, and desmoglein-3 (DSG3). These have become standard components in diagnostic panels, especially in cases with ambiguous morphology or limited squamous features. This is illustrated in Figure 2, which shows a case of moderately differentiated SCC with classic histologic features, diffuse p40 nuclear staining, and negative p16 expression.

The p40 antibody, which binds specifically to the ΔNp63 isoform of the p63 protein, is considered the most specific marker for squamous differentiation. Although both p40 and p63 are highly sensitive in detecting squamous carcinomas, p40 offers superior specificity, minimizing false positives in non-squamous tumors. For example, in pulmonary SCC, p40 stained 100% of cases, without cross-reactivity, in large B-cell lymphomas and with only occasional staining in adenocarcinomas [12]. In spindle cell SCC of the head and neck, p40 retained ~82% sensitivity in the sarcomatoid component, with patchy nuclear staining [4,9,10,11,12,13,14,15,16,17,18,19,20,21,22,23,24,25].

p63, while equally sensitive, displayed broader expression across both squamous and non-squamous neoplasms, including myoepithelial tumors and certain lymphomas. Thus, while not as specific, p63 remains valuable when interpreted within a panel, especially due to its diffuse and strong nuclear staining pattern in most SCC cases, including basaloid and clear cell variants [13,23,25,26,27,28].

Cytokeratin 5/6 (CK5/6), which identifies basal-type keratin expression, typically displays strong cytoplasmic staining in both conventional and variant forms of SCC. This marker remains detectable even in sarcomatoid and basaloid subtypes, and its expression is generally absent or limited in adenocarcinomas, enhancing its diagnostic utility [13,29,30].

Desmoglein-3 (DSG3), a desmosomal adhesion protein, was noted in several studies as a sensitive and complementary marker, with membranous staining in SCC nests. In esophageal SCC, DSG3 was positive in 100% of cases and proved especially useful in well-differentiated tumors with weak or absent p40 expression [13,31,32,33,34,35,36,37].

A comparative summary of marker performance is provided below (Table 1):

These markers retained diagnostic value across SCC variants:

- Basaloid SCC showed strong p63 and CK5/6 expression, aiding distinction from adenoid cystic carcinoma [39,40,41].
- Sarcomatoid SCC demonstrated focal positivity for p63/p40 and AE1/AE3 in the spindle component, confirming epithelial origin [9,24,25,28].
- Clear cell SCC expressed p63, p40, and CK5/6, though some studies noted decreased p40 intensity in clear areas [13,23,28,30].

In summary, a panel-based approach—typically combining p40 and CK5/6, optionally with p63 or DSG3—ensures the highest diagnostic accuracy in both conventional and variant SCC.

### 3.2. Markers Used in Differential Diagnosis

The immunohistochemical distinction between squamous cell carcinoma (SCC) and its histologic mimics is a critical step in diagnostic accuracy, especially in small biopsies or poorly differentiated tumors. Based on the analyzed studies, a consistent set of “exclusion markers” was employed to differentiate SCC from morphologically overlapping entities such as basal cell carcinoma (BCC), adenocarcinoma, and melanoma [42].

One of the most reliable markers for this purpose is Ber-EP4, an epithelial cell adhesion molecule. SCC is consistently negative for Ber-EP4, whereas BCC shows strong membranous positivity, making this marker particularly valuable in the differential diagnosis of cutaneous tumors. In documented cases of collision tumors, Ber-EP4 selectively marked the BCC component, while the adjacent SCC remained negative [14].

Epithelial membrane antigen (EMA), a general epithelial marker, is typically positive in SCC and negative in BCC, further supporting their distinction when p63 is positive in both. EMA is especially helpful in basaloid variants of SCC, where it can differentiate SCC from p63-positive myoepithelial tumors, which are usually EMA-negative [26].

In differentiating SCC from adenocarcinoma, especially in the lung and salivary glands, CK7, TTF-1, and napsin A serve as key markers. SCC usually lacks expression of CK7, while adenocarcinomas are CK7-positive, often with co-expression of TTF-1 and napsin A. A CK7−/p40+ profile strongly supports SCC, whereas CK7+/TTF-1+ suggests glandular origin [19,30,38].

TTF-1 (thyroid transcription factor-1) and napsin A are highly specific for lung adenocarcinomas and are negative in SCC. Their expression, especially when paired with CK7, is used to exclude non-squamous pulmonary neoplasms in thoracic pathology [12,18,19,20,30].

To exclude melanoma, especially amelanotic or spindle cell variants, S100 and SOX10 are indispensable. Both are negative in SCC, including sarcomatoid variants, but strongly positive in melanocytic tumors. Their use is essential in the evaluation of undifferentiated or spindle cell tumors to avoid misclassification [9,15,43,44,45].

A comparative table based on the reviewed literature is presented below (Table 2):

In several reports, these markers were used effectively in challenging contexts:

- In a poorly differentiated spindle cell tumor, negativity for S100 and SOX10 combined with patchy p63 and pan-cytokeratin positivity confirmed a diagnosis of sarcomatoid squamous cell carcinoma, excluding melanoma or sarcoma [9,15,43,47].
- In a maxillary gingival lesion suspected of BCC, Ber-EP4 positivity defined the BCC component, while EMA positivity and Ber-EP4 negativity in adjacent nests confirmed SCC [14,48].

**Figure 3:**
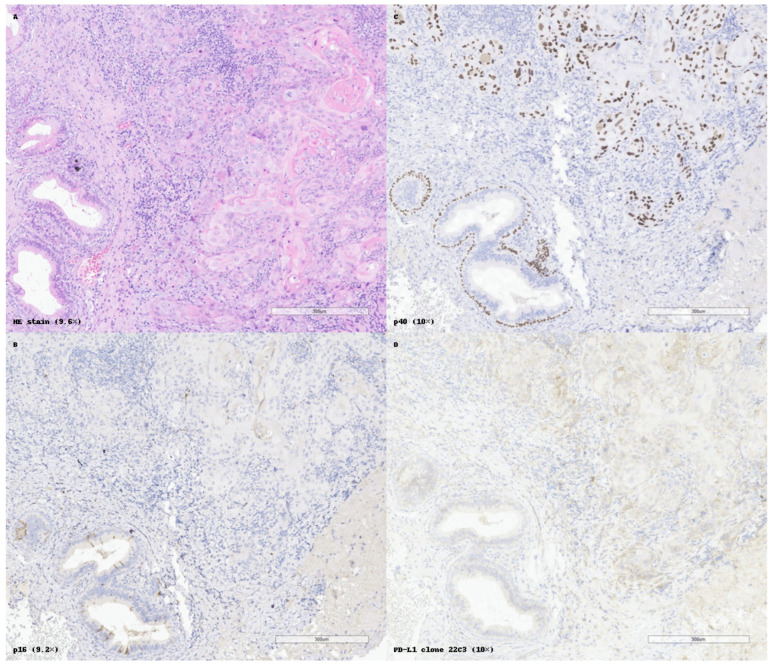
Histopathological and immunohistochemical features of a keratinizing squamous cell carcinoma (SCC). (**A**) Hematoxylin and eosin (HE) staining shows irregular anastomosing nests and cords of well-differentiated tumor cells with keratin pearl formation and abundant eosinophilic cytoplasm. (**B**) p16 immunostaining is entirely negative in tumor cells, indicating the absence of transcriptionally active HPV involvement. (**C**) Diffuse and strong nuclear positivity for p40 confirms squamous differentiation. The myoepithelial layer around some salivary ducts (internal control) is also stained. (**D**) PD-L1 immunohistochemistry using clone 22C3 reveals low to moderate membranous staining, suggestive of limited immunogenic potential in this case. All panels are presented at original magnifications ranging from 9.2× to 10×, as indicated.

Figure 3 exemplifies a well-differentiated keratinizing SCC with classical histology, diffuse p40 positivity, p16 negativity, and low membranous PD-L1 expression, consistent with limited immunotherapeutic potential.

Taken together, this exclusion-based panel approach enhances diagnostic specificity and prevents major diagnostic pitfalls. While markers like p40 and CK5/6 confirm squamous lineage, it is the negative staining for lineage-specific mimics (melanocytic, adnexal, or glandular) that ensures diagnostic precision.

### 3.3. Prognostic and Staging-Related Markers

Beyond their utility in confirming squamous differentiation, several immunohistochemical (IHC) markers have demonstrated value in assessing proliferative activity, invasiveness, and tumor aggressiveness in SCC. These biomarkers complement traditional TNM staging by providing functional and molecular data, thereby supporting more nuanced patient stratification.

The most widely studied marker in this context is Ki-67, a nuclear protein expressed during active phases of the cell cycle. High Ki-67 labeling index correlates with advanced tumor stage, poor histologic differentiation, and reduced overall survival, especially in oral and cervical SCC [1]. In dysplastic mucosa, elevated Ki-67 levels have also been associated with a greater risk of progression to invasive carcinoma, underscoring its relevance in early-stage assessment [17,49,50,51,52,53,54,55].

In mucosal SCCs, such as those in the cervix and oropharynx, p16 functions as an indirect indicator of high-risk HPV involvement. Its widespread, intense nuclear and cytoplasmic expression is often associated with more advanced disease, including regional lymph node spread and an aggressive clinical course in HPV-associated cancers. However, in non-HPV-related SCC, p16 expression is variable and less clearly prognostic [1,17,50,51,53].

CD44, a cell-surface glycoprotein, plays a role in both cell adhesion and the epithelial–mesenchymal transition. Its expression progressively increases from normal tissue to dysplastic changes and finally to invasive SCC, where it has been linked to enhanced invasiveness, metastatic behavior, and potential resistance to treatment. Co-expression of CD44 with TGF-β has been proposed as an indicator of malignant transformation risk in oral premalignant lesions [1,2,56,57,58].

Another prognostically relevant marker is xCT (SLC7A11), a cystine/glutamate antiporter that regulates oxidative stress responses. In oral SCC, high xCT expression—particularly when co-expressed with CD44—was associated with chemoresistance and poor clinical outcomes. This phenotype defines a subpopulation of tumor cells with enhanced survival capacity under oxidative stress [56,59].

Additional emerging markers include ARL4C and YAP, which have been investigated in rare histological subtypes such as clear cell SCC. Loss of expression of these regulators has been associated with dedifferentiation, more aggressive histology, and poor prognosis, although current evidence remains limited to small studies or case series [10,60,61,62].

These findings highlight the growing relevance of IHC not only in confirming diagnosis but also in informing prognosis and guiding management. Though not yet standardized across all pathology protocols, these markers show promise for integration into future clinical staging systems and risk-based treatment models.

### 3.4. Role of IHC in Early Detection and Lesion Stratification

Several studies have demonstrated that IHC is useful not only in diagnosing overt SCC but also in characterizing premalignant lesions and identifying those with higher malignant transformation risk. For instance, CD44 expression has been reported to increase progressively from normal epithelium to dysplasia and invasive SCC, with the most intense and diffuse staining seen in high-grade lesions [2,56,57]. This supports its role as a biomarker for early carcinogenic progression, particularly in oral leukoplakia or erythroplakia.

Similarly, Ki-67 is frequently used to assess epithelial proliferation. A high Ki-67 index in dysplastic mucosa correlates with increased likelihood of progression to SCC. The combination of Ki-67 and p16 is especially informative in HPV-related cervical or oropharyngeal premalignant lesions, where strong dual positivity signals viral integration and higher-grade intraepithelial neoplasia [1,16,17,54,63,64].

These applications demonstrate that IHC, when applied early, can support screening, risk stratification, and biopsy triage in lesions that may not yet meet the morphological criteria for carcinoma.

## 4. Discussion

This review provides a comprehensive synthesis of the most frequently used immunohistochemical (IHC) markers in the diagnosis and staging of squamous cell carcinoma (SCC), offering a practical, structured overview of their diagnostic and prognostic roles. The data extracted from 91 peer-reviewed articles reveal a clear trend: IHC has become indispensable in the accurate identification and subclassification of SCC, particularly in cases with ambiguous histology or poorly differentiated features.

### 4.1. Diagnostic Value of IHC in SCC

The findings of this review reinforce the importance of specific immunohistochemical markers in identifying squamous cell carcinoma and its variants. p40, in particular, stands out for its exceptional nuclear specificity and sensitivity, not only in conventional SCC but also in less-differentiated forms such as sarcomatoid and basaloid subtypes. Notably, p40 maintains immunoreactivity in sarcomatoid areas where other epithelial markers may be reduced, making it a key tool for confirming epithelial lineage in tumors with spindle morphology. Although p63 remains highly sensitive, its broader expression in non-squamous neoplasms—such as myoepithelial tumors and lymphomas—limits its diagnostic specificity. This supports the current consensus favoring p40 over p63 for SCC confirmation. Nevertheless, p63 retains value within a panel due to its robust nuclear staining and widespread expression across SCC subtypes, including clear cell and basaloid variants [4,9,12,19,22,28,39].

As a marker of basal keratin expression, CK5/6 typically exhibits strong cytoplasmic staining in both well-differentiated and poorly differentiated squamous tumors. Its utility is especially pronounced in cases where nuclear markers are inconsistently expressed, such as minimally keratinized tumors. Moreover, its specificity is relatively high, with limited staining observed in adenocarcinomas [9,13,65,66,67].

DSG3 enhances the diagnostic panel by providing distinct membranous staining, particularly along cell junctions in tumor nests. It is especially valuable in well-differentiated SCCs where other markers, such as p40 or p63, may be less intensely expressed. This makes DSG3 a valuable adjunct in cases with preserved architecture but weak nuclear reactivity [13,31,36,68].

The combination of these markers in a standardized panel allows for more confident diagnostic classification, especially in small biopsies, incisional samples, or cases with overlapping morphology. Importantly, histologic variants of SCC—including sarcomatoid, basaloid, and clear cell forms—retain sufficient expression of these markers to permit accurate immunophenotypic confirmation, although intensity and distribution may vary.

### 4.2. IHC in Differential Diagnosis: Avoiding Pitfalls

An important aspect highlighted by this review is the utility of immunohistochemical analysis in differentiating SCC from tumors with similar histological features. This is especially crucial when the tumor morphology is unclear or the degree of differentiation is limited, making histopathological evaluation alone insufficient [14]. This was particularly evident in reported “collision tumors”, where IHC permitted clear separation of dual tumor populations.

In pulmonary or salivary gland pathology, the challenge often lies in differentiating SCC from adenocarcinoma. Here, a panel including CK7, TTF-1, and napsin A is critical: these markers are generally absent in SCC but positive in adenocarcinomas, especially of lung origin. A CK7−/p40+ profile strongly supports squamous differentiation, while CK7+/TTF-1+ favors a glandular phenotype [20,38,46,65,69,70].

Another group of neoplasms that may resemble SCC includes amelanotic melanomas and spindle cell tumors. In such cases, the application of melanocytic markers like S100 and SOX10 is critical. These markers are typically not expressed in SCC, including its sarcomatoid forms, but are characteristically present in melanomas, thereby facilitating accurate exclusion [9,45,47,71].

### 4.3. Prognostic and Staging Relevance

Beyond diagnosis, IHC has demonstrated increasing utility in assessing tumor biology and staging parameters. The Ki-67 index serves as an important measure of tumor proliferation and has been associated with more advanced disease and unfavorable clinical outcomes. In HPV-driven mucosal SCCs, p16 expression, originally used as a proxy for viral presence, has also been linked to regional lymph node metastasis and more aggressive tumor behavior [1]. HPV-independent cervical SCCs are frequently diagnosed at an advanced stage, and they have a higher rate of lymph node metastasis, which confers a reduced disease-free and overall survival [72,73,74,75].

CD44, as a marker of cancer stemness and EMT, and xCT, related to redox metabolism and chemoresistance, identify high-risk SCC subpopulations that may require more aggressive treatment strategies. These findings suggest that IHC is not only diagnostic but stratificational, with real potential to impact patient management when incorporated into staging systems [2,56,76,77].

**Figure 4:**
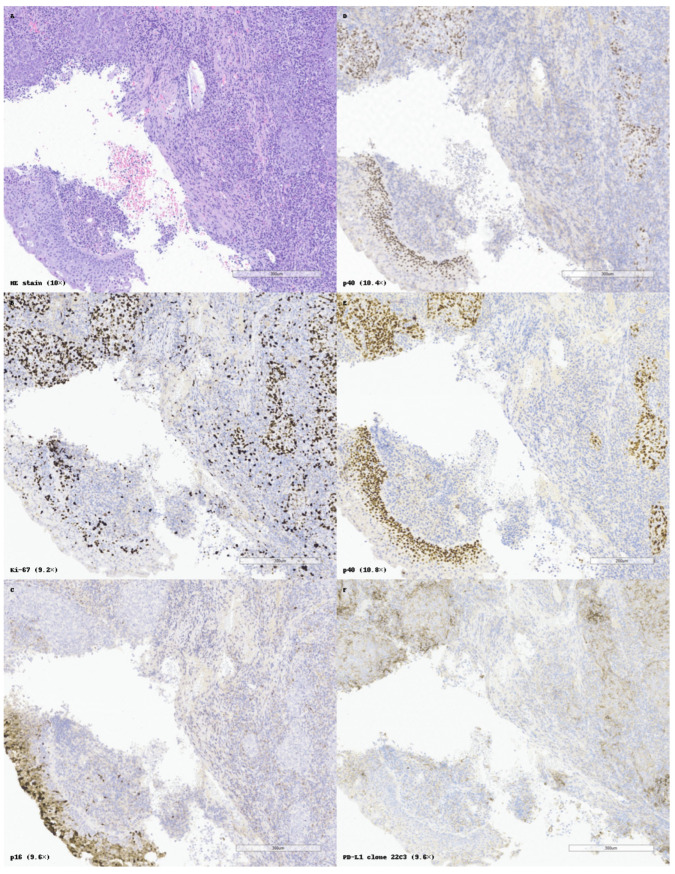
Histopathological and immunohistochemical profile of a poorly differentiated non-keratinizing squamous cell carcinoma (SCC) of the nasopharynx. (**A**) Hematoxylin and eosin (HE) staining shows compact nests of basaloid tumor cells with scant cytoplasm, high nuclear-to-cytoplasmic ratio, and no keratinization. (**B**) Ki-67 immunostaining highlights intense nuclear proliferation in most tumor cells, reflecting a high mitotic index. Internal control in basal cells from the adjacent intact squamous epithelium. (**C**) p16 shows focal and weak nuclear and cytoplasmic positivity, raising the possibility of HPV involvement but without strong diagnostic support. (**D**) Nuclear staining for p40 shows limited distribution, marking only a subset of tumor cells. (**E**) A second p40 clone demonstrates broader and more intense nuclear expression, strengthening the diagnosis of squamous differentiation. (**F**) PD-L1 (clone 22C3) reveals heterogeneous membranous expression in tumor cells, indicating potential immunotherapy responsiveness. All panels are presented at original magnifications ranging from 9.2× to 10.8×, as indicated.

Immune checkpoint molecules, particularly PD-L1 (programmed death-ligand 1), have also emerged as clinically significant in non-keratinizing nasopharyngeal SCC. Immunohistochemical detection using the 22C3 clone, which highlights membranous PD-L1 expression, is now routinely applied to evaluate eligibility for checkpoint blockade therapy. A 2024 meta-analysis by Xu et al. demonstrated that PD-L1 positivity (≥1%) in nasopharyngeal carcinoma correlates with improved response rates and progression-free survival under anti-PD-1/PD-L1 therapy. This supports the role of PD-L1 as a predictive and biologically stratifying biomarker, especially in advanced-stage or recurrent disease [78,79,80,81]. This pattern is exemplified in Figure 4, which illustrates a poorly differentiated non-keratinizing SCC of the nasopharynx, with high Ki-67 labeling index, absence of p16 expression, and diffuse but heterogeneous PD-L1 positivity.

Notably, even in rare variants such as clear cell SCC, expression of ARL4C and YAP provided clues to dedifferentiation and clinical behavior, expanding the utility of IHC beyond traditional morphology-based grading [10].

### 4.4. Toward Standardized Interpretation and Panels

**Figure 5:**
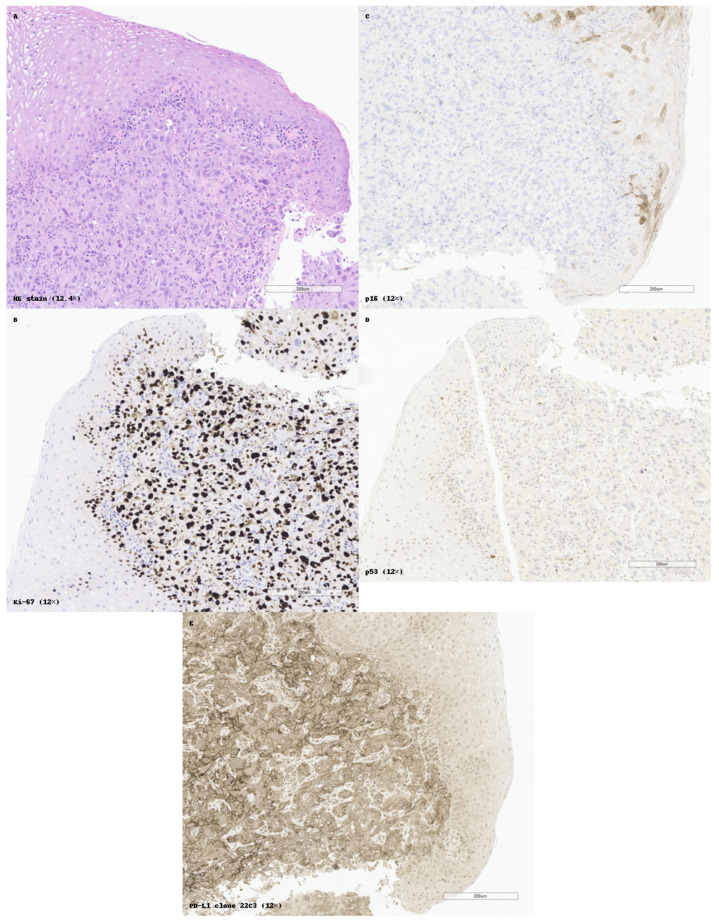
Histopathological and immunohistochemical features of a squamous cell carcinoma (SCC) of the epiglottis. (**A**) Hematoxylin and eosin (HE) staining showing invasive anastomosing tumor nests without keratinization and marked nuclear atypia. (**B**) Ki-67 immunostaining reveals a high proliferative index, with numerous positively labeled tumor cell nuclei. (**C**) p16 shows negative to focal nuclear and cytoplasmic expression, suggesting a non-HPV-driven oncogenic pathway. (**D**) Normal “Wild type” expression of p53. (**E**) PD-L1 immunohistochemistry using clone 22C3 reveals strong membranous staining in tumor cells, supporting eligibility for immune checkpoint inhibitor therapy. All panels are shown at original magnifications ranging from 12× to 12.4×, as indicated.

Figure 5 illustrates a practical example of standardized IHC application, with p16/Ki-67 dual interpretation, PD-L1 assessment, and internal controls supporting diagnostic accuracy in SCC of the epiglottis. Despite the availability of numerous immunohistochemical (IHC) markers for squamous cell carcinoma (SCC), lack of standardization in marker selection and interpretation remains a challenge in daily diagnostic practice. One of the key findings of this review is the variability with which markers are applied across institutions and anatomical sites, often depending on individual pathologist preference, tissue availability, or clinical context.

A standardized panel-based approach, tailored to site and morphology, could significantly improve both diagnostic consistency and clinical relevance. For example:

- In pulmonary SCC, the minimal diagnostic panel often includes p40, CK5/6, and TTF-1, allowing reliable differentiation from adenocarcinoma [12,30].
- In head and neck lesions, especially where basaloid features are present, a combination of p63 or p40, EMA, and Ber-EP4 can reliably separate SCC from adnexal tumors or BCC [14,46].
- In spindle cell or undifferentiated tumors, the addition of S100 and SOX10 is crucial to exclude melanoma, while Ki-67 and pan-cytokeratin confirm epithelial lineage and assess proliferation [9,45,71].

These examples support the idea that marker interpretation should be algorithmic and context-driven, rather than fragmentary. This also includes an awareness of marker limitations—for instance, p63 cross-reactivity in myoepithelial tumors or CK5/6 expression in some basal-type adenocarcinomas—which underscores the need for multi-marker strategies and internal controls [30,66,67,82].

An additional approach that may enhance diagnostic precision and tissue economy is the use of dual immunohistochemical stains. By combining two markers within the same tissue section—such as p40/napsin A or CK5/6/TTF-1—dual staining enables simultaneous interpretation based on subcellular localization (e.g., nuclear vs. cytoplasmic) and reduces tissue consumption. In a comparative study involving 58 NSCLC cases, dual-marker staining achieved 100% concordance with single-marker protocols in identifying SCC and adenocarcinoma, while offering a time-saving and cost-efficient workflow—particularly in limited biopsy material [18,30,83,84,85,86].

Another well-established application of dual staining is in HPV-related squamous neoplasia, where p16/Ki-67 co-expression has demonstrated strong correlation with high-grade intraepithelial lesions. In cervical cytology, this dual stain significantly improves specificity and positive predictive value compared to cytology or HPV DNA testing alone. In a study of 93 women, dual p16/Ki-67 positivity was strongly associated with CIN2+ histology, supporting its use in triage protocols to reduce unnecessary colposcopies and overtreatment in young patients with low-grade findings [16,17,63,87,88,89,90].

Moreover, the lack of uniform scoring systems for many prognostic markers (e.g., Ki-67, p16, CD44) limits reproducibility across studies. Adoption of semi-quantitative tools such as the H-score, Allred score, or defined positivity thresholds could enhance both diagnostic and research utility.

This review contributes by synthesizing a framework in which diagnostic, differential, and prognostic markers are organized by function and clinical use. Future work should focus on prospective validation of standardized panels, ideally stratified by anatomical site, histologic variant, and molecular subtype. This would facilitate the broader incorporation of IHC into multidimensional staging and personalized care models.

### 4.5. Quality Control and Technical Solutions in Conventional IHC

While immunohistochemistry has become central to SCC diagnostics, its effectiveness depends on high technical quality and consistent interpretation. Several challenges persist in conventional IHC practice, particularly in relation to sample integrity, incomplete staining, and interpretive variability. Suboptimal fixation (e.g., under- or over-fixation) may result in epitope degradation or masking, leading to weak or false-negative staining, especially for labile antigens like Ki-67 or PD-L1 [91,92]. Similarly, inadequate antigen retrieval or improper antibody titration can lead to in-consistent results, affecting reproducibility across laboratories.

To address these issues, standardized protocols for pre-analytical handling—including immediate fixation in 10% neutral-buffered formalin for 6–72 h—are strongly recommended [93,94]. The use of internal positive controls within the same histological section (e.g., non-neoplastic basal cells or inflammatory infiltrates) is critical to confirm staining reliability and detect technical failure [95]. All IHC stains included in this review were performed on formalin-fixed, paraffin-embedded tissues using validated protocols and included appropriate internal controls. Automated IHC platforms have also demonstrated improved consistency by minimizing user variability, offering precise control over incubation, reagent distribution, and slide handling [96,97].

Another key development is the integration of quality assurance programs (QAPs), such as external quality assessments (EQA) and digital slide archiving for remote peer validation. Studies have shown that participation in such programs significantly reduces diagnostic discrepancies and helps identify recurrent technical flaws [98,99].

Lastly, digital pathology tools have started to play an increasing role in monitoring staining completeness and uniformity. Image analysis software can detect under-stained areas, quantify marker intensity, and identify artifacts or edge effects—thereby enhancing slide-level quality assurance and reducing interpretive error [100,101].

Addressing these practical factors is essential to ensure that IHC provides not only diagnostic information but also reproducible, actionable results across laboratories and patient cohorts.

### 4.6. The Potential Role of Digital Immunohistochemistry in SCC

The interpretation of immunohistochemical markers in squamous cell carcinoma (SCC) is often subjective and prone to interobserver variability, especially for markers with complex or borderline expression patterns, such as PD-L1. In recent years, digital immunohistochemistry (digital IHC) has gained increasing attention as a strategy to improve standardization, reproducibility, and quantification in IHC evaluation [102].

Digital IHC employs whole slide imaging combined with artificial intelligence or algorithm-based analysis to objectively assess marker expression. Several studies have shown that digital platforms can improve the consistency of scoring for PD-L1, Ki-67, and p53, particularly in tumors with heterogeneous staining or challenging morphology. Moreover, it offers the possibility of remote consultation and consensus scoring, which is relevant for rare tumor subtypes or borderline IHC results [103].

Despite these advantages, digital IHC still faces important limitations, including variable validation of algorithms, high implementation costs, and limited integration into routine pathology workflows. Nevertheless, its future role in SCC diagnosis and biomarker-based therapeutic decisions appears promising, especially in the context of increasing use of PD-L1 immunotherapy and standardized reporting [104].

## 5. Limitations

While this review provides a structured synthesis of immunohistochemical (IHC) markers in squamous cell carcinoma (SCC), several limitations must be acknowledged with respect to methodology, data heterogeneity, and generalizability of the findings.

### 5.1. Narrative Design and Absence of Quantitative Synthesis

This work was designed as a narrative, literature-based review, rather than a fully systematic review with meta-analysis. As a result, the sensitivity and specificity values discussed were derived from individual studies and interpreted qualitatively, without pooled statistical analysis.

Additionally, several of the included studies were descriptive in nature—including case reports and small case series—which may not accurately reflect population-level trends. The lack of standardized IHC scoring systems (e.g., Allred score, H-score, or defined cut-offs) in many reports limited cross-study comparisons of staining intensity and diagnostic thresholds.

### 5.2. Heterogeneity of Tumor Sites and Antibody Clones

SCC arises in a variety of anatomical locations, each with distinct molecular and etiological features. This review included data from cutaneous, oral, esophageal, laryngeal, cervical, and pulmonary SCCs. While this broad inclusion improves generalizability, it also introduces biological heterogeneity, particularly in relation to HPV-driven vs. HPV-independent tumorigenesis.

In addition, differences in antibody clones and staining protocols across studies (e.g., p63 clones 4A4 vs. 7JUL; CK5/6 clones D5/16B4 vs. MA5-12596) may affect reproducibility. Many reports did not specify the antibody clones used, antigen retrieval methods, or dilution parameters, further complicating the interpretation of inter-study variability.

### 5.3. Limited Representation of Rare Variants

While the review included several articles discussing variant histologies of SCC (e.g., basaloid, sarcomatoid, clear cell), these were often limited to single case reports or small series, making it difficult to draw firm conclusions regarding IHC marker performance in these subtypes. The rarity of these variants inherently limits the evidence base, and future larger studies or registries would be needed to validate the diagnostic strategies proposed here.

### 5.4. Lack of Outcome Correlation in Many Studies

Although markers such as Ki-67, CD44, and p16 have recognized prognostic value, many studies reviewed here did not include clinical follow-up or survival analysis. As such, conclusions about the predictive or staging value of these markers were drawn primarily from associative data. This limits the ability to integrate IHC findings into evidence-based treatment algorithms or prognostic models, which would require prospective validation in larger cohorts.

### 5.5. Potential Publication Bias

Finally, as with any literature-based review, there is a risk of publication bias, whereby studies reporting positive or confirmatory results are more likely to be published and indexed. Negative findings or studies with ambiguous IHC expression patterns may be underrepresented, skewing the perceived utility of some markers. Moreover, the review did not include unpublished studies, abstracts, or gray literature, which may omit emerging data from recent conference presentations or ongoing trials.

Despite these limitations, the structured synthesis of available data provides practical, pathology-focused insights into the use of IHC in SCC and highlights the need for standardization and prospective validation of marker panels in diverse clinical contexts.

## 6. Conclusions

Squamous cell carcinoma (SCC) represents a morphologically and biologically diverse group of epithelial malignancies with wide anatomical distribution and variable clinical behavior. In this context, immunohistochemistry (IHC) has emerged as an indispensable tool, enabling not only accurate tumor identification but also histological subtyping and risk stratification, even in small or poorly differentiated specimens.

This review consolidates current evidence regarding the most frequently used IHC markers in SCC, highlighting their role in positive diagnosis, differential diagnosis, and prognostic assessment. By organizing the data across functional and clinical categories, this synthesis offers a practical and structured reference for pathologists, oncologists, and trainees involved in SCC diagnostics.

Although this review is not comprehensive of all available markers, it emphasizes the increasing importance of IHC in current cancer diagnostics. The adoption of marker panels tailored to tumor subtype and site can enhance accuracy, minimize diagnostic ambiguity, and help guide therapeutic decisions, even in uncommon forms of SCC.

### Future Directions

Based on the current synthesis, several key areas for future research and clinical integration can be identified:

- Prospective validation of IHC panels across different SCC variants and anatomical sites, with standardized cut-offs and scoring systems;
- Development of semi-quantitative frameworks (e.g., Allred or H-score) for prognostic markers such as Ki-67, CD44, and p16;
- Integration of IHC with molecular tools, including next-generation sequencing, transcriptomic profiling, or digital pathology platforms, to enhance personalized diagnostics;
- Establishment of treatment algorithms that incorporate IHC-based stratification into routine clinical decision-making, especially in HPV-related and chemoresistant SCCs;
- Exploration of real-time or non-invasive diagnostic methods, such as digital immunostaining, ex vivo confocal microscopy, or liquid biopsy correlates of IHC markers.

As IHC continues to evolve, its full potential lies in its integration—not only with histological assessment, but also with molecular tools, in the service of precision medicine.

In conclusion, immunohistochemistry remains the cornerstone of modern SCC diagnosis. When used in a targeted and systematic manner, IHC not only confirms tumor identity but also opens the door to biologically informed patient care. Its full potential lies in its integration not only with morphology but with molecular tools and clinical data, in the service of precision oncology.

## Figures and Tables

**Table 1:** Diagnostic performance and staining patterns of commonly used IHC markers in cutaneous SCC.

Marker	Sensitivity in SCC	Specificity for SCC	Staining Pattern	Notes
p40	95–100%	~98–100%	Strong nuclear	Most specific for SCC [12]
p63	97–100%	~60–80%	Nuclear	Broader expression; less specific [38]
CK5/6	90–100%	~95–98%	Cytoplasmic	Retained in sarcomatoid, basaloid variants [13]
DSG3	~98–100%	~90–95%	Membranous (cell–cell borders)	Useful in well-differentiated SCC [13]

**Table 2:** Comparative immunohistochemical profiles of SCC and histologic mimics in differential diagnosis.

Marker	SCC	BCC	Melanoma	Adenocarcinoma	Diagnostic Role
p40	+	+ (often)	−	−	Sensitive but not exclusive to SCC [13]
Ber-EP4	−	+	−	+	Distinguishes SCC (−) from BCC (+) [14,46]
EMA	+	−	−	+	Helps separate SCC from BCC [26]
CK7	−	−	−	+	Adenocarcinoma marker [38]
TTF-1	−	−	−	+	Lung adenocarcinoma marker [12]
S100	−	−	+	−	Melanocytic marker [9,15]
SOX10	−	−	+	−	Specific for neural crest origin [9,47]

## Data Availability

No new data were created or analyzed in this study. Data sharing is not applicable to this article.
